# Evaluation of Diagnostic Performance of Line Probe Assay on Smear-Negative Samples of Pulmonary Tuberculosis

**DOI:** 10.7759/cureus.58298

**Published:** 2024-04-15

**Authors:** Sumi Nandwani, Sanjay Singhal, Dipti Gothi, Ritu Singhal, Naz Perween, Kavita Gupta, Raunak Bir, Jayanthi Gunasekaran, Anushka Soni, Urmila Chaudhary

**Affiliations:** 1 Microbiology, Post Graduate Institute of Child Health, Noida, IND; 2 Microbiology, Employee's State Insurance Corporation (ESIC) Hospital, Postgraduate Institute of Medical Sciences and Research (PGIMSR), New Delhi, IND; 3 Pulmonary and Critical Care Medicine, Employee's State Insurance Corporation (ESIC) Hospital, Postgraduate Institute of Medical Sciences and Research (PGIMSR), New Delhi, IND; 4 Microbiology, WHO National Reference Laboratory and Center of Excellence (TB) and National Institute of Tuberculosis and Respiratory Diseases, New Delhi, IND

**Keywords:** smear-negative samples, first-line drug sensitivity testing, acid-fast bacilli, diagnostic utility of lpa, mycobacterium tuberculosis complex detection, ziehl-neelsen stain, isoniazid resistance, rifampicin resistance, line probe assay, pulmonary tuberculosis

## Abstract

Background: This study aims to compare the performance of line probe assay (LPA) on smear-negative samples with that of smear-positive samples for diagnosing pulmonary tuberculosis (PTB) and first-line drug sensitivity testing (FL DST).

Methods: A total of 196 sputum samples including both smear-positive (112) and negative (84) samples of patients suspected of PTB were subjected to LPA for TB detection and FL DST. TB culture followed by MPT 64 Ag was done and conventional FL DST was performed on all culture-positive isolates. Results of LPA on smear-negative were compared with smear-positive samples.

Results: The LPA confirmed the diagnosis of PTB in 104/112 smear-positive cases but in only 36/84 smear-negative cases. The assay had 47.36%, 72.72%, and 88.88% sensitivity and 86.96%, 95.23%, and 95.65% specificity in smear-negative cases compared to 89.09%, 95.83%, and 98.07% sensitivity and 100%, 98.36%, and 98.24% specificity in smear-positive cases for detecting *Mycobacterium tuberculosis* (MTB), rifampicin (RMP) resistance, and isoniazid (INH) resistance, respectively.

Conclusion: LPA performance was better on smear-positive than smear-negative sputum samples. Further larger studies are needed to justify the use of LPA on smear-negative pulmonary samples for diagnosis.

## Introduction

Globally, it is estimated that 10.6 million people developed tuberculosis (TB) disease, and there were 1.3 million deaths from TB among HIV-positive people in 2022. The continuous spread of multidrug-resistant tuberculosis (MDR-TB) and extensively drug-resistant tuberculosis (XDR-TB) is a major challenge for global TB control. Globally, 7.5 million people were diagnosed with TB, among them 410000 cases were identified with MDR-TB in 2022. In 2022, eight countries accounted for 58% of global MDR-TB cases including India (27%) [1].

Traditional culture and drug-susceptibility testing (DST) using solid media can require eight to 12 weeks to yield results [2]. However, faster liquid-based culture techniques provide results within four to six weeks. Unfortunately, these liquid methods are prone to high contamination rates, which restrict their practicality [3]. The currently available conventional methods lead to delays in DST and can lead to prolonged periods of therapy and ongoing transmission of the disease. The development of rapid molecular diagnostic tests for the identification of *Mycobacterium tuberculosis *(MTB) and Drug Resistance has consequently become a research and implementation priority [4].

In 2008, the World Health Organization (WHO) officially certified and endorsed the line probe assay (LPA) as a molecular method for the rapid diagnosis of TB. This assay also enables the simultaneous detection of resistance to two crucial anti-TB drugs: rifampicin (RMP) and isoniazid (INH) [5]. The LPA is built upon DNA-STRIP technology, which allows for efficient and accurate detection of specific genetic markers associated with TB and drug resistance. This advancement has significantly improved the speed and precision of TB diagnosis, benefiting patients and healthcare systems worldwide [6]. Although LPA needs more technical expertise and takes longer to perform than the Xpert MTB/resistance to rifampin (RIF) assay (Cepheid, USA), it has the ability to detect both low-level and high-level INH and RMP resistance unlike Xpert MTB/RIF assay [7]. The newer LPA version 2 is able to detect MTB and DST patterns for the drugs, RMP and INH, in smear-positive and smear-negative samples and culture-positive isolates [8].

In our study, we aimed to evaluate the diagnostic performance of LPA version 2 on smear-negative samples compared to smear-positive samples of suspected PTB cases to (a) detect MTB (diagnose TB) and (b) to detect resistance against first-line drugs, RMP and INH, in culture-positive MTB cases.

## Materials and methods

Ethical approval of the study was granted by the Institutional Ethical Committee (DM(A)H-19/16/15/2012-PGIMSR). Patients (All age groups with suspected pulmonary tuberculosis (PTB) or those receiving retreatment) were recruited after obtaining written informed consent. The sample size required of a total of 196 sputum samples from presumptive MDR-TB patients was calculated based on a non-probability convenience sampling technique with a 95% confidence interval and 5% alpha error, assuming a 97% sensitivity of the assay [9].

Inclusion criteria

(a) All the patients suspected of PTB of all age groups. (b) All the PTB patients suspected of having drug resistance to either or both RMP and INH. (c) All the patients whose samples had adequate quantities for testing and proper labeling. (d) All the patients whose clinical and radiological records were available. 

Exclusion criteria

(a) Samples that do not have proper labeling and do not have adequate quantity. (b) All the patients with samples with no clinical and radiological details.

Sample collection

Sputum samples were collected in sterile containers as per guidelines from 196 consenting patients [10].

Sample processing

All sputum samples were microscopically examined by Ziehl-Neelsen (ZN) staining and followed by decontamination by the NALC-NaOH method [11]. After decontamination, the samples were processed for TB culture and LPA.

Tuberculosis Culture

A 0.5 mL processed sample was introduced into the BacT-Alert tube and placed in an automated BacT-Alert instrument. The incubation occurred at 37˚C for a maximum duration of 42 days, with regular monitoring [12]. The time to positivity was documented for the tubes flagged as positive, which were subsequently confirmed by using ZN staining for AFB followed by immunochromatographic test (ICT) test for MPT-64 Ag (SD Bioline, Standard Diagnostics, Suwon, Korea) to rule out non-tuberculous mycobacteria (NTM) and confirm MTB [13].

Drug Sensitivity Test

All culture-positive isolates, which were identified as MTB (MPT-64 Ag positive), were tested for resistance to first-line TB drugs by using conventional phenotypic DST in Lowenstein-Jensen solid media with a 1% proportion method. The concentrations for testing RMP and INH were 40 mg/mL and 0.2 mg/mL, respectively. Any strain with 1% of bacilli resistant to any of the two drugs, RMP and INH, was classified as resistant to that drug [14].

Line Probe Assay

Mycobacterial DNA was extracted using a GenoLyse kit (Hain Lifescience, Nehren, Germany)-based manual method. PCR was performed using pre-made amplification mixes. Reverse hybridization was done using a GT Blot 48 device (Hain Lifescience, Nehren, Germany), and the results were interpreted according to the manufacturer's instructions. Results of LPA on smear-negative and smear-positive samples were compared for (a) detection of MTB in all samples and (b) detection of RMP and INH resistance only in samples that were culture-positive for MTB. Both composite reference standard (CRS) and mycobacterial cultures were used to evaluate the results of LPA on direct samples for the detection of MTB cases in this study. CRS included a combination of clinical symptoms, radiological findings, TB culture result, other bacterial culture results, clinical response of patients to anti-tuberculosis treatment (ATT), or other antibiotic treatment/cartridge-based nucleic acid amplification test (CBNAAT) results [15,16].

Based on a CRS algorithm the patients were categorized as follows: Group A, TB cases: clinical suspicion of individuals who are having TB based on clinical symptoms and signs with evidence suggestive of TB culture-positive/TB culture-negative with other evidence suggestive of TB-like radiological findings consistent with TB, negative results for other bacterial infections on culture, non-response to antibiotics (other than ATT), and response to ATT after two months of being positive. Group B: non-TB cases: clinically suspected of TB but no other evidence for TB, i.e., either TB culture negative, radiological features not suggestive of TB, bacterial culture-positive for any other bacteria, or the patient’s response to antibiotic (other than ATT) treatment.

To evaluate LPA results on smear-negative and smear-positive samples for detection of RMP and INH resistance, only samples that were TB culture-positive were included, and phenotypic DST on culture-positive isolates was taken as a gold standard.

To minimize bias, all laboratory technicians were blinded to respective laboratory results. Any discrepant results were confirmed by the National Reference Laboratory at National Institute of Tuberculosis and Respiratory Diseases (NITRD), Delhi.

Statistical analysis

Statistical analysis was conducted using SPSS version 29 software packages for evaluating the Genotype MTBDRplus version 2 LPA. Various parameters like sensitivity, specificity, positive predictive value, and negative predictive value were calculated among all samples, comparing the results with the reference standard method. CRS was used as the reference standard for the detection of TB, and conventional DST was taken as the gold standard to evaluate resistance to RMP and INH. Results of LPA for both smear-negative and smear-positive samples were calculated and compared.

Comparative analysis of the performance of LPA was done on smear-negative versus smear-positive sputum samples for (a) detection of MTB using a comprehensive reference standard as a gold standard, (b) diagnosis of RIF resistance using conventional DST as the gold standard, and (c) diagnosis of INH resistance using conventional DST as the gold standard.

## Results

Samples of a total of 196 patients with 122 males (62.24%) and 74 females (37.76%) were evaluated in the study. The mean age of patients was 33.44 years. Of these 112 were smear-positive (57.14%) and 84 were smear-negative (42.86%). Results were that a total of 148 patients were classified as TB cases and 48 were classified as non-TB cases based on CRS. For the detection of MTB, LPA demonstrated an overall sensitivity of 78.37%, specificity of 87.5%, PPV of 95.08%, and NPV of 56.75% on directly tested specimens. Our analysis demonstrated that the assay performed much better in smear-positive samples (sensitivity: 89.09%; specificity: 100%; PPV: 100%) compared to smear-negative samples (sensitivity: 47.36%; specificity: 86.96%; PP: 75%) for detection of MTB infection except for a better NPV in smear-negative samples (66.66%) than in smear-positive samples (14.28%) (Table 1).

**Table 1:** Results of LPA for detection of PTB CRS, composite reference standard; TB, tuberculosis; MTB, *Mycobacterium tuberculosis*; LPA, line probe assay; NPV, negative predictive value; PPV, positive predictive value pulmonary tuberculosis

LPA version 2		Results			Performance indicator for LPA			
		TB cases (on the basis of CRS)	Non-TB cases (on the basis of CRS)	Total	Sensitivity (%)	Specificity (%)	PPV (%)	NPV (%)
All samples	MTB positive	117	6	122	78.37	87.5	95.08	56.75
	MTB negative	32	42	74				
	Total	148	48	196				
Smear-positive samples	MTB positive	98	0	98	89.09	100	100	14.28
	MTB negative	12	2	14				
	Total	110	2	112				
Smear-negative samples	MTB positive	18	6	24	47.36	86.96	75	66.66
	MTB negative	20	40	60				
	Total	38	46	84				

A total of 143 samples were TB culture-positive of which two were found to be atypical mycobacteria (MPT: 64 Ag negative). For the detection of RIF and INH resistance, only 141 samples that were TB culture-positive were evaluated, and LPA results on smear-positive (109) and smear-negative (32) samples were evaluated with phenotypic conventional drug sensitivity performed on culture-positive isolates as the gold standard. Any discrepant or indeterminate results were confirmed by testing at NITRD. Results as in Table 2 and Table 3 were of 141 culture-positive MTB isolates; by conventional DST, 59 (41.80%) were found to be MDR TB (RIF and INH resistant), 61 (43.26%) were found to be INH resistant (two were found to be mono INH resistant). There was no case of mono RIF resistance.

**Table 2:** Results of LPA for detection of RIF resistance RMP, rifampicin; LPA, line probe assay; LPA, line probe assay; NPV, negative predictive value; PPV, positive predictive value; S, sensitive; R, resistant

LPA version 2		Results			Performance indicator for LPA			
		RIF resistance	RIF sensitive	Total	Sensitivity (%)	Specificity (%)	PPV (%)	NPV (%)
All samples	RIF R	54	2	56	91.5	97.56	96.42	94.11
	RIF S	5	80	85				
	Total	59	82	141				
Smear-positive samples	RIF R	46	1	47	95.83	98.36	97.87	96.77
	RIF S	2	60	62				
	Total	48	61	109				
Smear-negative samples	RIF R	8	1	9	72.72	95.23	88.88	86.95
	RIF S	3	20	23				
	Total	11	21	32				

**Table 3:** Results of LPA for detection of INH resistance INH, isoniazid; LPA, line probe assay; NPV, negative predictive value; PPV, positive predictive value; S, sensitive; R, resistant

LPA version 2		Results			Performance indicator for LPA			
		INH resistance	INH sensitive	Total	Sensitivity (%)	Specificity (%)	PPV (%)	NPV (%)
All samples	INH R	59	2	61	96.72	97.5	96.72	97.5
	INH S	2	78	80				
	Total	61	80	141				
Smear-positive samples	INH R	51	1	52	98.07	98.24	98.07	98.24
	INH S	1	56	57				
	Total	52	57	109				
Smear-negative samples	INH R	8	1	9	88.88	95.65	88.88	95.65
	INH S	1	22	23				
	Total	9	23	32				

LPA on direct samples demonstrated an overall sensitivity of 91.5%, specificity of 97.56%, PPV of 96.42%, and NPV of 94.11% for detection of RIF resistance and a sensitivity of 96.72%, specificity of 97.5%, PPV of 96.72%, and NPV of 97.5% for the detection of INH resistance. However, results of LPA on smear-positive samples (sensitivity: 95.83%, specificity: 98.36%, PPV: 97.87%, and NPV: 96.77% for the detection of RIF resistance; sensitivity: 98.07%, specificity: 98.24%, PPV: 98.07%, and NPV: 98.24% for detection of INH resistance) was much better than smear-negative samples (sensitivity: 72.72%, specificity: 95.23%, PPV: 88.88%, NPV: 86.95% for detection of RIF resistance as well as sensitivity: 88.88%, specificity: 95.65%, PPV: 88.88%, NPV: 86.95% for detection of INH resistance) as shown in Table 2 and Table 3.

59/141 (41.84%) samples were detected as RIF resistant, of which 54 were also detected as RIF resistant by LPA. All RIF-ce. The most common mutation in resistant cases was MDR TB; there was no case of mono RIF resistance. A most common mutation in case of RIF resistance was found to be missing of wild type 8 (codons 530-533) and associated MUT3, i.e., S531L amino acid substitution (n=43, 79.6%), followed by codons 526-529-WT7 (n=4, 7.4%), codons 513-519-WT3/4 (n=3, 5.5%), D517V-MUT1 (n=1, 1.8%), codons 510-517 WT2/3 (n=1, 1.8%), codons 510-513-WT2 (n=1, 1.8%), and codons 505-509-WT1 (n=1, 1.8%). 61/141 (43.26%) strains were INH resistant (2 cases of mono INH resistance) of which 59 were detected as INH resistant by LPA; 52 (88.13%) were missing wild type (315) and mutation S315TL in katG gene, which was the most frequent mutation associated with INH resistance; four were inhA resistant, and three were both katG and inhA resistant.

## Discussion

In our study of evaluation, the performance of genotype MTBDRplus version 2 LPA was higher for the detection of MTB in smear-positive sputum samples (sensitivity: 89.09% and specificity: 100%) compared to smear-negative samples (Sensitivity: 47.36% and specificity: 86.96%). Despite the challenges posed by smear-negative sputum samples, some studies have highlighted the favorable sensitivity and specificity of the genotype MTBDRplus version 2 LPA in detecting MTB [17,18]. However, most studies have reported findings similar to ours, i.e., higher sensitivity and specificity of LPA version 2 for the detection of MTB in smear-positive sputum samples compared to smear-negative samples [19,20]. A meta-analytical study reported a pooled sensitivity of LPA version 2. of 94% (89.4-99.4%) for smear-positive specimens and 44% (20.2-71.7%) for smear-negative specimens for MTB detection [5]. Yadav et al., 2013 and 2021, reported that the majority of invalid results from the genotype MTBDRplus version 2 LPA were observed in sputum specimens with lower bacillary load (1+) or culture-negative samples [21,22]. Meaza et al. (2017) in Ethiopia reported 77.8% sensitivity of genotype MTBDRplus version 2 LPA for the detection of MTB from smear-negative sputum samples and found a high level of invalid results for the detection of MTB [9]. Studies on LPA version 1 have reported lower detection rates of MTB in smear-negative specimens [5]. This could be because LPA version 1 uses mechanical methods for MTB genomic DNA isolation, unlike the chemical method with the Genolyse kit in LPA version 2, which decreases the chance of cross-contamination and increases the quality of the amplified genomic DNA, producing better results.

Our study revealed an overall sensitivity of 91.5% and a specificity of 97.6% for the detection of RIF resistance on direct samples. A meta-analysis by Ling et al., 2008, in Canada, reported a pooled sensitivity of 98.1% and specificity of 98.7%, while Nathavitharana et al., 2017, in Boston, USA, reported a pooled sensitivity of 96.3% and specificity of 98.2% for RIF resistance of HAIN version 2 on direct samples [5,23]. In our study, LPA version 2 had a higher sensitivity and specificity for the detection of RIF resistance in smear-positive samples (sensitivity: 95.83%, specificity: 98.36%, PPV: 97.87%, and NPV: 96.77%) compared to smear-negative samples (sensitivity: 72.72%, specificity: 95.23%, PPV: 88.68%, and NPV: 86.95%). Some studies have reported a reasonably good sensitivity of 100% and specificity of 99.24% of LPA version 2 for the detection of RIF resistance in smear-negative samples [9]. Nathavitharana et al., 2017, in Boston, USA, have reported that LPA version 2 has performed poorly in the detection of RIF resistance on smear-negative samples [5]. 

In our study, LPA version 2 was found to have an overall sensitivity of 96.72% and specificity of 97.5% for the detection of the INH resistance on direct samples, which was better than the results of a meta-analysis by Nathavitharana et al., 2017, which reported a pooled sensitivity of LPA version 2 on direct samples to be 89.2% and specificity of 98.4% for detection of INH resistance on direct samples [5]. This may be because we have evaluated only culture-positive isolates. In our study, LPA version 2 was found to have lower performance (sensitivity: 88.88% and specificity: 95.65%) in smear-negative samples than in smear-positive samples (sensitivity: 98.07% and specificity: 98.24%) for detection of INH resistance. Most other studies have also reported a better performance of LPA version 2 for detection of INH resistance in smear-positive samples than in smear-negative samples like our results. Meaza et al., 2017, in Ethiopia, reported a sensitivity of 97.2%, specificity of 91.7%, PPV of 97.1%, and NPV of 92.1%, respectively, for the detection of INH resistance directly from smear-positive sputum sample but a low sensitivity of 60% for the detection of INH resistance directly from smear-negative and culture-positive sputum sample of genotype MTBDRplus version 2 LPA [9]. However, Singh et al., 2017, has reported a high sensitivity of 97.6% and specificity of 98.6%, of LPA version 2 for detection of INH resistance in smear-negative samples in India [17]. In our study, some cases of mono INH resistance were detected by LPA version 2. Similar results have been reported in another study [17]. In our study, 41.84% of samples were RIF resistant, which is similar to the other study [9]. The most common mutation in the case of RMP resistance was found to be missing wild type 8 (530-533) and MUT3, i.e., S531L amino acid substitution, which is similar to that reported in other studies [5,9,23]. In our study, 43.26% of strains were INH resistant and the most frequent mutation associated with INH resistance was missing wild type (315) and mutation S315TL in the katG gene, which has also been reported in other studies [24,25]. In our study, three falsely RMP-sensitive results and one false positive INH-sensitive result have been detected by LPA version 2 and this could be due to the fact that about 5% and 10% to 25% of resistant strains are thought to have mutations outside rpoB, katG, and inhA loci, respectively [9,24,25]. The possible reason for a few false negative results could have been due to some masked mutations in the genomic regions of rpoB, inhA, and katG genes [25].

The limitation of our study is that LPA is based on DNA detection so it might end up detecting even the treated and the latent cases not necessarily the active cases of TB. Due to the limited sample size in our study, extrapolation of our results on the general population would require further studies on a larger sample size. 

## Conclusions

The genotype MTBDRplus version 2 LPA demonstrated high sensitivity and specificity in detecting MTB from direct smear-positive sputum samples. However, its performance in direct smear-negative sputum samples was suboptimal, with a notable proportion of invalid results. However, in cases of MTB* *detected by LPA, the sensitivity and specificity of detecting resistance to RMP and INH were similar to other studies. LPA has a limiting factor of not detecting resistance from lower bacterial load samples but it is a highly useful test in settings where there is no facility of Truenat, CBNAAT, or liquid culture system for detection and drug resistance.
